# Orphans of the HIV epidemic: the challenges from toddlerhood to adolescence and beyond

**DOI:** 10.7448/IAS.17.4.19483

**Published:** 2014-11-02

**Authors:** Mamatha M Lala

**Affiliations:** 1 Pediatric Ward, Wadia Group and Hospital, Mumbai, India; 2 Mumbai Smiles, Mumbai, India

## Abstract

This presentation focuses on the challenges and practical issues faced each day by orphans of the HIV epidemic and the holistic care that can be provided, as they continue to grow from toddlerhood to adolescence and beyond. An HIV Research Trust Scholarship enabled me to spend quality time in a sub-Saharan African province worst hit by the HIV epidemic and to interact with local experts and learn from mutual clinical experience. It was an immensely useful exercise as the clinical spectra of the diseases are very similar to ours and they have ongoing active research programs very relevant to our setting. India is arguably home to the largest number of orphans of the HIV epidemic. The responsibility of caring for orphaned children overwhelms and pushes many extended families beyond their ability to cope. Many countries are experiencing large increases in the number of families headed by women and grandparents, or even young children. These households are often unable to meet basic needs, and so the number of children living on the streets is rising. Orphaned children are disadvantaged in many devastating ways. In addition to the trauma of witnessing the sickness and death of one or both parents and perhaps siblings, they lack the necessary parental guidance through crucial life-stages of identity formation and transition into adulthood. They are more likely to suffer damage to their cognitive and emotional development and be subjected to; exploitation in terms of labour, social exclusion, extreme economic uncertainty, physical and sexual abuse, illiteracy, malnutrition and illness. Education remains a distant dream. With stigma and discrimination, they lack legal protection, lose inheritance rights, access to essential services available to other community members and professional help from doctors, teachers and lawyers. The implications for these unfortunate children are extraordinarily grave but governments, international agencies, non-governmental organizations, schools, other community groups and individuals can still alter the course of the crisis. The Committed Communities Development Trust (CCDT) is a voluntary secular Trust, reaching out to 300,000 people annually, focusing intensively on children affected and infected by HIV/AIDS, mainly orphans, child headed families, children living in street situations, brothels, institutions and children at risk of drug addiction, abuse and exploitation in Mumbai. We run several comprehensive HIV/AIDS programmes addressing issues of prevention, care, support, education, awareness, empowerment, training and research through strongly structured home-based care programs, community based programs and temporary residential shelters. The CCDT recognizes and understands the daunting challenges these children face and helps them overcome these as a team by providing comprehensive care and support, giving them an opportunity in life and enabling them to become productive citizens of tomorrow.

